# Combination Therapy with Indigo and Indirubin for Ulcerative Colitis via Reinforcing Intestinal Barrier Function

**DOI:** 10.1155/2023/2894695

**Published:** 2023-02-14

**Authors:** Jin Xie, Shimin Tian, Jun Liu, Shengjie Huang, Ming Yang, Xiangbo Yang, Runchun Xu, Junzhi Lin, Li Han, Dingkun Zhang

**Affiliations:** ^1^State Key Laboratory of Characteristic Chinese Drug Resources in Southwest China, Chengdu University of Traditional Chinese Medicine, Chengdu 611137, China; ^2^Key Laboratory of Modern Preparation of TCM, Ministry of Education, Jiangxi University of Traditional Chinese Medicine, Nanchang 330004, China; ^3^State Key Laboratory of Innovation Medicine and High Efficiency and Energy Saving Pharmaceutical Equipment, Jiangxi University of Traditional Chinese Medicine, Nanchang 330004, China; ^4^Yaan Xunkang Pharmaceutical Co., Ltd., Yaan 625600, China; ^5^TCM Regulating Metabolic Diseases Key Laboratory of Sichuan Province, Hospital of Chengdu University of Traditional Chinese Medicine, Chengdu 610072, China

## Abstract

Indigo and indirubin, the active molecules of traditional Chinese medicine indigo naturalis, exert therapeutic activity for ulcerative colitis (UC). Indigo and indirubin are isomers and have distinctive profiles in anti-inflammation, immune regulation, intestinal microbiota regulation, oxidative stress regulation, and intestinal mucosal repair for UC treatment. Thus, exploring its combined administration's integrated advantages for UC is critical. This study is aimed at clarifying the effect and mechanisms of the combined administration of indigo and indirubin on colitis mouse models. The results showed that all the treatment groups could improve the disease symptoms, and the combined administration showed the best effect. Additionally, compared with indigo and indirubin alone, the combination group could significantly reinforce intestinal barrier function by increasing the expression of E-cadherin, occludin, ZO-1, and MUC2 and improving intestinal permeability. The treatment groups significantly improved the expression of cytokines, including TNF-*α*, IFN-*γ*, IL-12, IL-23, and IL-17A, and indirubin presented the most potent anti-inflammatory effect. Furthermore, all the treatment groups reduced the infiltration of the immune cells in intestinal lamina propria and the production of ROS/RNS. Notably, indigo exhibited a more substantial capacity to regulate natural killer (NK) cells, ILC3, neutrophils, and dendritic cells, followed by the combination group and indirubin alone. Finally, all the treatment groups modulated intestinal microbiota composition, increased the proportion of beneficial microbiota, and decreased the proportion of microbiota. Our results indicated that indigo and indirubin synergistically reinforced the intestinal barrier function, which may be associated with integrating the indirubin anti-inflammatory and intestinal microbiota regulating strength and indigo immune and ROS/RNS regulation advantage.

## 1. Introduction

Ulcerative colitis (UC) is a persistent inflammatory bowel disease (IBD) mainly caused by the injury of the colorectal mucosa and submucosa, which gradually spreads to the whole colon and can damage the ileocecal part [1]. UC is characterized by persistent or recurrent diarrhea, mucus abscess, and blood stool with abdominal pain, tenesmus, and varying degrees of systemic symptoms and is affected by many factors such as heredity [2], immune system imbalance [3, 4], intestinal barrier dysfunction, and microbial disorder [5, 6]. In the past decades, UC has become a worldwide public health challenge for its increasing incidence rate and the difficulties in curing it [7]. Although much progress has been made in the diagnosis and therapy of UC in recent years, availability of an effective therapeutic drug is still challenging [8]. Thus, novel, efficient, and safe therapeutic strategies for treating UC are needed. The gut is a physiological symbiotic system composed of epithelial cells, mucus layers, intestinal microbiota, and immune cells that promote mucosal immune tolerance, nutrient cycling, and defense functions. The intestinal environment primarily affects the mucosal barrier, including immune cells, inflammation, and intestinal microbiota. During UC pathology, neutrophils, macrophages, innate immune cells, and natural killer (NK) cells aggregate and infiltrate into the colon tissue, leading to the release of inflammatory factors and chemokines, such as TNF-*α*, IFN-*γ*, and IL-22, resulting in low expression of tight junction proteins and an impaired intestinal mucosal barrier, which in turn affects intestinal permeability [9–11]. Innate lymphoid cells (ILCs) develop from typical lymphoid progenitor cells (CLP) and resemble adaptive lymphocytes in morphology [12]. It is considered an essential regulator of tissue homeostasis and inflammation by releasing cytokines, including IL-22, IL-17, and granulocyte macrophage-colony stimulating factor (GM-CSF), to protect the intestinal mucosa from infection by various pathogens, maintain intestinal homeostasis, and further affect the intestinal mucosal barrier of UC [13]. For example, IL-22 prevents bacterial infections, relieves intestinal inflammation, and restores tissue injury during hepatitis or colitis [14]. IL-17 can stimulate epithelial and endothelial cells to secrete chemokines and other chemoattractants and can also influence the inflammatory immune response by recruiting proinflammatory neutrophils. It can also maintain and protect the intestinal mucosal epithelial barrier by independently regulating the tight junction protein [15, 16]. IL-1*β* and IL-23 can be released by macrophages and dendritic cells (DCs) under stimulation of microbes, which might keep the conversion of ILC1 to the natural cytotoxicity receptor (NCR+) ILC3 subset, contributing to epithelial barrier integrity and proliferation, stimulating the secretion of AMPs, REG3*γ*, and mucin, and enhancing epithelial fucosylation [13]. Moreover, the excessive generation of reactive oxygen species (ROS) and reactive nitrogen species (RNS) produced by neutrophils and other immune cells could disrupt intestinal mucosal barrier function [17, 18]. In addition, dysregulation of the intestinal microbiome under UC may decrease beneficial bacteria and increase harmful bacteria, resulting in the imbalance of an intestinal mucosal barrier. For example, the beneficial bacteria *Lactobacillus* may decompose amino acid tryptophan into aryl hydrocarbon receptor (AHR) ligands through the IL-22-mediated intestinal mucosal response [19]. *Bifidobacterium* species strengthen the intestinal mucus layer through autophagy and calcium signaling, enhancing the intestinal mucosal barrier [20]. Bacteria such as *Escherichia coli* can affect the expression of tight junction proteins and increase intestinal permeability, damaging the intestinal mucosa [21]. Consequently, the microbiome and the immune system influence the intestinal mucosal barrier, which may work together to regulate UC. Indigo naturalis is clinically effective in treating UC. The Japanese scholars Shinya Sugimoto et al. conducted an open-label prospective study in which 20 patients with moderate UC activity were included. The capsules containing indigo naturalis were given orally at 2 g twice daily for eight weeks. After treatment, the overall clinical efficacy was 72%, the overall clinical remission rate was 33%, and the mucosal healing rate was 61% [22]. In another multicenter, double-blind trial conducted in Japan, 86 patients with active UC (Mayo score) were included. The rates of mucosal healing in patients who received placebo and 0.5, 1.0, and 2.0 g of IN daily were 13.6%, 56.5%, 60.0%, and 47.6%, respectively. This study further demonstrates the favorable efficacy of indigo naturalis in treating patients with UC. This clinical evidence indicated that the clinical positioning of indigo naturalis for UC has advantages in the remission of inflammation, especially in repairing the intestinal mucosa. According to the European Crohn's and Colitis Organisation (ECCO) treatment guidelines for UC, the rate of mucosal healing is the primary evaluation index for the clinical efficacy of UC [23]. Therefore, intestinal mucosal repair is the key to treating ulcerative colitis.

Indigo (IND) and indirubin (INB) are the main pharmacodynamic components of indigo naturalis, which are isomers of each other and have their characteristics in the treatment of UC. INB has anti-inflammatory advantages. In the dextran sodium sulfate- (DSS-) induced colitis mouse model, INB treatment could inhibit body weight loss and improve DAI score. INB can significantly decrease the expression of TNF-*α*, IFN-*γ*, IL-2, IL-4, and IL-10 [24]. Furthermore, INB could suppress the lipopolysaccharide-induced inflammation via TLR4 abrogation mediated by the NF-*κ*B and MAPK signaling pathways [25]. IND, a ligand for aryl hydrocarbon receptor (Ahr), is advantageous in immune and intestinal microbiota regulation. IND could improve body weight loss and shorten the colon length of DSS-induced colitis. Further, the population of splenic CD4^+^ IL-10^+^ T cells could be increased by IND [26]. Another research showed that IND could ameliorate DSS-induced colitis by modulating the intestinal microbiota community, primarily by increasing the abundance of probiotics (*Lactobacillus*) and reducing the abundance of harmful bacteria (*Streptococcus*) [27], and thus may regulate the intestinal mucosal barrier. Additionally, INB and IND could mitigate colonic oxidative damage. In the DSS-induced UC mice, INB and IND could significantly reduce the production of myeloperoxidase (MPO) [27]. Another research showed that INB could reduce malondialdehyde (MDA) levels, superoxide dismutase (SOD) activity, and glutathione (GSH) levels in DSS-induced UC mice [28]. Indigo and indirubin are both present in indigo naturalis, and indigo naturalis improves UC through synergistic effects of various mechanisms. The effect of indigo naturalis in the treatment of UC is higher than that of pure INB and IND, which reveals that INB may play a synergistic role with IND. Therefore, the combination of INB and IND may play a role in the overall control of the intestinal mucosal barrier via anti-inflammation, immune regulation, oxidative stress, and intestinal microbiota regulation. There is a lack of systematic research on the intestinal mucosal barrier, intestinal microbiota, oxidative stress, and immune cells in treating UC with IND and INB. Simultaneously, the difference in the effect of IND and INB in UC treatment and the advantages of their combined application have not been studied.

Consequently, this paper is aimed at investigating the efficacy of the combined administration of IND and INB in UC treatment and elucidating the mechanism of their combined administration from the perspective of repairing the intestinal mucosal barrier.

## 2. Materials and Methods

### 2.1. Chemicals and Reagents

IND (≥98% purity) was purchased from Chengdu Keloma Co., Ltd. INB (≥98% purity) was purchased from Chengdu Pufeide Biotechnology Co., Ltd. Dextran sulfate sodium (DSS, 40 kDa) was purchased from Xibao Biotechnology Co., Ltd. Other chemicals, solvents, and reagents were all analytical.

### 2.2. Animals

All animal studies in this article are reported in compliance with the Animal Research: Reporting of In Vivo Experiments (ARRIVE) guidelines. Male Balb/C mice (20-22 g) were provided by Beijing Sibford (Permit No. SYXK (Chuan) 2019-049). All the studies were carried out following the National Institutes of Health Guide for the Care and Use of Laboratory Animals. Mouse experiments were approved by the Animal Ethics Committee, Chengdu University of Traditional Chinese Medicine (ethics approval number: 2021-49). Mice were housed under specific pathogen-free conditions on a standard 12/12 h light/dark cycle at 25 (Permit No. SYXK (Chuan) 2019-049). All mice were given free access to food and water and a week to acclimate at our facility.

### 2.3. Induction of Acute DSS Colitis and Experimental Design

For 10 days, mice were fed 3% DSS in drinking water to induce colitis, except for the normal group. After a 1-week acclimation period under the controlled environment, all mice were randomly assigned to the following groups (*n* = 8/group): normal group (distilled water every day), DSS group (3% DSS for 10 days), IND group (20 mg/kg IND for 10 days), INB group (20 mg/kg INB for 10 days), and combination group (17.64 mg/kg IND and 2.35 mg/kg INB for 10 days). The body weight change and disease activity index (DAI) scores were monitored daily, including body weight, gross bleeding, and stool consistency. By the end of the treatment, mice were sacrificed by intraperitoneal injection of pentobarbital, the colons were removed, and the colon length was recorded.

### 2.4. Histological Analysis Assessment of Cytokines

The distal colon tissue fragments were fixed in 4% paraformaldehyde (*w*/*v*), paraffin-embedded, sectioned at 3 *μ*m, and stained with hematoxylin. Briefly, the severity of inflammation was determined by the following criteria: 0, none; 1, mild inflammation to the mucosa and submucosa; 2, moderate inflammation to the lamina muscularis propria; 3, moderate inflammation and the crypt is wholly gone; and 4, marked infiltration in the mucosa and submucosa in deeper layers and loss of crypts structure. Images were taken with a Nikon Eclipse E100 microscope. To observe the mucus in colonic tissues, Alcian blue staining was performed. Briefly, the sections were stained with Alcian blue and Nuclear Fast Red and rinsed with tap water until colorless. Tissue sections were subjected to Periodic Acid-Schiff (PAS)/hematoxylin under a light microscope to visualize the goblet cells. Cytokine levels in colonic tissue homogenates were measured by mouse TNF-*α*, IFN-*γ*, IL-12, IL-23, and IL-17A ELISA kits (Elabscience, Wuhan, China) according to the manufacturer's instructions.

### 2.5. In Vivo Living Imaging and Intestinal Permeability Measurement

The in vivo live imaging of the mice was performed using a Multifunctional Imaging System (IVIS Spectrum, PerkinElmer). The production of reactive oxygen and nitrogen species ROS/RNS was detected via the probe L-012 (Wako, Japan) sodium-mediated chemiluminescence one day before the end of the experiment, as previously described [29]. The mice were immobilized using the anesthetic isoflurane (1.5-2.5%) and injected intraperitoneally with L-012 (25 mg/kg). The automated exposure setup was used to capture the bioluminescence images. The intestinal permeability was evaluated by the in vivo imaging fluorescence of chemical fluorescein isothiocyanate- (FITC-) dextran (Sigma). Briefly, the abdominal regions of the mice were shaved to eliminate the influence of autofluorescence and light scattering caused by fur before the experiments. Then, the mice were fasted overnight and gavaged with FITC-dextran (600 mg/kg). After four hours, the mice were anesthetized with isoflurane and placed in the supine position on nonfluorescent black paper on the imaging platform before being imaged at 480 nm excitation and 520 nm emission. The fluorescence intensities were analyzed with the software of a live imaging system. Furthermore, for each mouse, the eyeball was removed from the socket to obtain the blood samples. The serum was obtained by centrifuging the whole blood at 6000 rpm for 15 min. A total of 50 *μ*L of supernatant was collected in a fresh tube and diluted with 100 *μ*L of PBS, then loaded into a 96-well black plate to measure the fluorescence intensity at 480 nm excitation and 520 nm emission with a fluorescence microplate reader (SpectraMax iD5, USA). FITC-dextran concentrations of serum were determined from standard curves generated by serial dilution.

### 2.6. Western Blot Analysis

Colon tissues were cut into tiny pieces, lysed in RIPA buffer (Servicebio, Wuhan, China, G2007-1ML), and disrupted with a Dounce homogenizer. The protein concentration was made uniform using the Pierce BCA protein assay kit (Biosharp, BL521A). Protein samples were separated by 10% SDS-polyacrylamide gel electrophoresis (SDS-PAGE) and transferred onto a polyvinylidene fluoride (PVDF) membrane. The membranes were washed twice with 1x TBS buffer and blocked with 1x TBST containing 5% nonfat milk for an hour at room temperature. The membranes were then left to incubate with specific primary antibodies overnight at 4°C. On the following day, the membranes were incubated with diluted secondary antibodies in TBST for an hour and then washed three times with TBST at room temperature for 15 min per wash. According to the manufacturer's instructions, ECL was used to visualize proteins which were then exposed to the ChemiDoc Gel Imaging System to obtain the images.

### 2.7. Immunohistochemistry and Immunofluorescence Measurement

Immunohistochemistry and immunofluorescence assay was used to identify the expression of E-cadherin, occludin, ZO-1, and MUC2. Paraffin-embedded colon sections were dewaxed and rehydrated before blocking to avoid endogenous peroxidase activity by 3% H_2_O_2_. For immunohistochemistry detection, colonic sections were incubated with primary antibodies against E-cadherin (Affinity, AF5145), occludin (Affinity, DF7504), ZO-1 (Affinity, AF5145), and MUC2 (Affinity, DF8390) overnight at 4°C, followed by incubating with streptavidin-horseradish peroxidase. The images were captured with a biological microscope (Olympus, CX21FS1). For immunofluorescent detection, the slices were treated overnight at 4°C with primary antibodies E-cadherin, occludin, and ZO-1. After washing with PBS three times to remove the extensive antibodies, the sections were incubated with secondary antibodies in the dark for an hour at room temperature. Lastly, the slides were incubated with DAPI solution (Wuhan Google Biotechnology Co., Ltd.), covered with a mounting medium, and examined under a fluorescence microscope (Nikon, Japan).

### 2.8. Quantitative Real-Time PCR

Real-time quantitative polymerase chain reaction (qPCR) was used to detect the mRNA expression of E-cadherin, occludin, ZO-1, and MUC2. Total RNA was extracted from tissues by the Animal Total RNA Isolation Kit (Foregene) and reverse transcribed into cDNA using a 5x All-In-One MasterMix (with AccuRT Genomic DNA Removal kit). The qPCR was performed with a K2800 automated nucleic acid sequence analyzer (Beijing Kaiao Technology Development Co., Ltd.) to quantify the mRNA. Supplementary Table 1 shows the primers used for PCR amplification. The 2^−△△Ct^ method was used to normalize the expression level of each gene to the GADPH expression.

### 2.9. Flow Cytometry Analysis

The colonic lamina propria cells were isolated using the methods previously reported by Godinho-Silva et al. [30]. To analyze neutrophils (CD11b^+^ Gr-1^+^), macrophages (CD11b^+^ F4/80^+^), and dendritic cells (CD11b^+^ CD11c^+^), the cell suspensions were stained with the following: PerCP/cyanine5.5 anti-mouse/human CD11b, PE anti-mouse CD11c antibody, APC anti-mouse F4/80 antibody, and FITC anti-mouse Ly-6G/Ly-6C (Gr-1) antibody. For NK cells (CD335, CD27, and CD11b, gated on CD3^–^NK1.1^+^), the cell suspensions were stained with PE/cyanine7 anti-mouse/rat/human CD27, PerCP/cyanine5.5 anti-mouse/human CD11b, PE anti-mouse CD161/NK1.1 antibody, FITC anti-mouse CD3 antibody, and APC anti-mouse CD335 antibody. For ILC1 (T-bet^+^) and ILC2 (GATA3^+^), the cell suspensions were stained with the following: ER780 anti-mouse CD45 antibody, FITC Ms CD3/Gr-1/CD11b/CD45R(B220)/Ter-119, PerCP/cyanine5.5 anti-mouse CD127 (IL-7R*α*), PE anti-T-bet, and anti-Hu/Mo Gata3. The antibodies for flow cytometry were obtained from Elabscience and BioLegend. Flow cytometry analysis was performed using a CytoFLEX flow cytometer (Beckman Coulter). Data were analyzed through the CytExpert software.

### 2.10. 16S rRNA Sequencing of Intestinal Microbiota

The fecal samples were kept at a low temperature with dry ice and were sent to Shanghai Meggie Biomedical Technology Co., Ltd. for 16S rRNA amplification of the V3-V4 region. The total DNA of fecal samples was extracted using an E.Z.N.A.® soil DNA kit (Omega Bio-tek, Norcross, GA, USA) according to the manufacturer's instructions. The DNA was checked by 1.0% agarose gel electrophoresis and quantitated on a NanoDrop® ND-2000 spectrophotometer (Thermo Scientific Inc., USA) [31]. The universal primers 338F (5′-ACTCCTACGGGAGGCAGCAG-3′) and 806R (5′-GGACTACHVGGGTWTCTAAT-3′) were used to amplify the V3-V4 region of the 16S rRNA gene [32]. The PCR reaction mixture includes 4 *μ*L of 5x FastPfu buffer, 2 *μ*L of 2.5 mM dNTPs, 0.8 *μ*L of each primer (5 *μ*M), 0.4 *μ*L of FastPfu polymerase, 10 ng of template DNA, and ddH2O to a final volume of 20 *μ*L. PCR amplification cycling conditions were as follows: initial denaturation at 95°C for 3 min, followed by 27 cycles of denaturing at 95°C for 30 s, annealing at 55°C for 30 s, extension at 72°C for 45 s, single extension at 72°C for 10 min, and end at 4°C. All samples were amplified in triplicate. The PCR product was extracted from 2% agarose gel and purified using the AxyPrep DNA Gel Extraction Kit (Axygen Biosciences, Union City, CA, USA) according to the manufacturer's instructions and quantified using a Quantus™ Fluorometer (Promega, USA). The Meiji biological cloud platform was used to conduct the data analysis, including species composition, species difference, and environmental correlation analysis.

### 2.11. Statistical Analysis

In all the experiments, the data were expressed as mean ± SEM. GraphPad Prism 5.0 software was used to draw and compare several groups using the one-way analysis of variance (ANOVA). *P* < 0.05 was considered statistically significant. ^∗^*P* < 0.05 and ^∗∗^*P* < 0.01, when compared with the DSS group; ^#^*P* < 0.05 and ^##^*P* < 0.01, when compared with the combination group.

## 3. Results

### 3.1. INB and IND Alleviated the Inflammatory Injury and ROS/RNS Production of DSS-Induced Colitis

**Figure 1:**
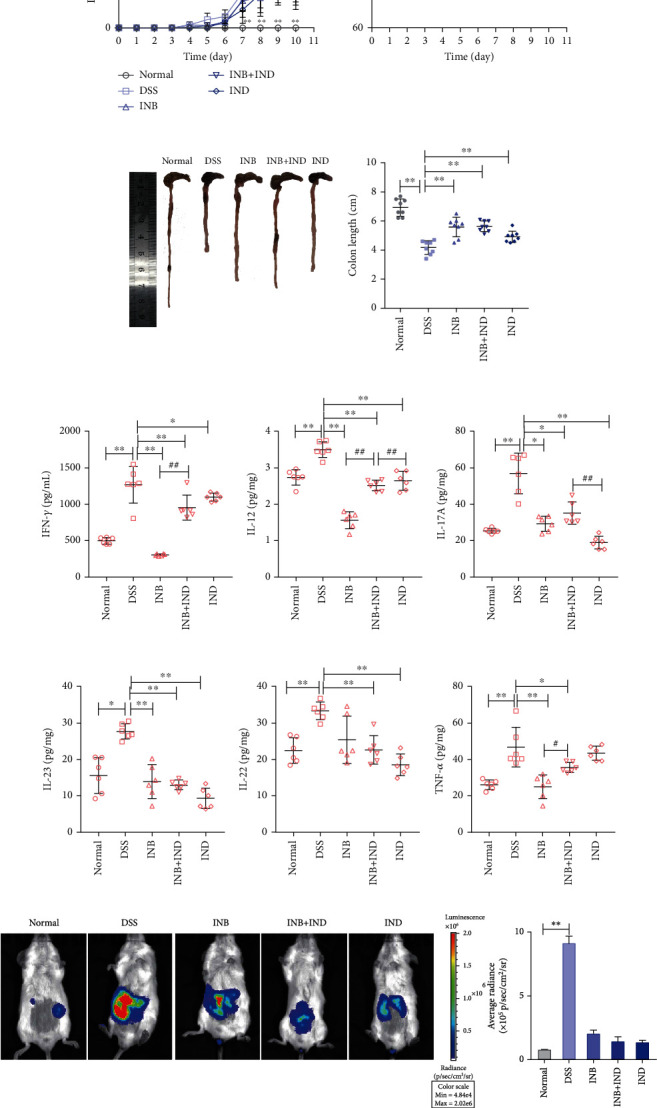
INB and IND alleviated the inflammatory injury of DSS-induced colitis. (a) DSS-induced UC mice were orally administrated with INB (20 mg/kg), IND (20 mg/kg), and INB+IND (20 mg/kg), and the normal mice were given normal drinking water throughout the experiment. (b) Body weight change (% initial body weight) and disease activity index (DAI) of mice, *n* = 8. (c) Representative images of colon morphology and statistics of colon length, *n* = 8. (d–i) Cytokine secretion in colon tissue, *n* = 6. (j) Representative images of bioluminescent imaging and ROS/RNS signals after administration of L-012, *n* = 3. (k) Representative images of H&E staining of proximal colon and histological scores. ^∗^*P* < 0.05 and ^∗∗^*P* < 0.01 compared with the DSS group; ^#^*P* < 0.05 and ^##^*P* < 0.01 compared with the INB+IND group, *n* = 3.

To explore the therapeutic effects of INB and IND on UC mice, the UC mouse model was established by supplementing 3% DSS in drinking water freely from the whole test period; INB (20 mg/kg), INB+IND (20 mg/kg), and IND (20 mg) were orally administrated daily for 10 days (Figure 1(a)). As illustrated in Figure 1(b), the body weight and DAI in the DSS group rapidly decreased and increased. In contrast, the INB and IND group decreased the DAI characterized by body weight, gross bleeding, and stool consistency. Furthermore, the colon length in the treated group was significantly longer than that in the DSS group (*P* < 0.01).

To determine the effect of INB and IND on the inflammatory colon tissues, we measured the levels of various cytokines by ELISA. As shown in Figures 1(d)–1(i), the treated group decreased the expression of inflammatory cytokines compared with the DSS group (*P* < 0.01). Moreover, the L-012-mediated luminescence in vivo was acquired to present the inflammation signals in mice. Consistently, the treated group alleviated the L-012 signal in vivo, indicating that the ROS/RNS production could be ameliorated following INB and IND treatment (Figure 1(j)). Moreover, the INB group appears to show a more potent anti-inflammatory effect than the other treatment group. Histologically, the DSS group showed significant inflammatory cell infiltration, damaged intestinal mucosal structures with focal villus edema, goblet cells, and crypt loss, while the treated group potently prevented colon tissue damage and inflammation scoring (Figure 1(k), *P* < 0.01). Notably, the result showed that the combination group could significantly reduce the histological scores than INB or IND alone. These results suggest that INB and IND reduced the development and severity of intestinal inflammation.

### 3.2. INB and IND Maintained the Intestinal Mucosal Physical Barrier Function

**Figure 2:**
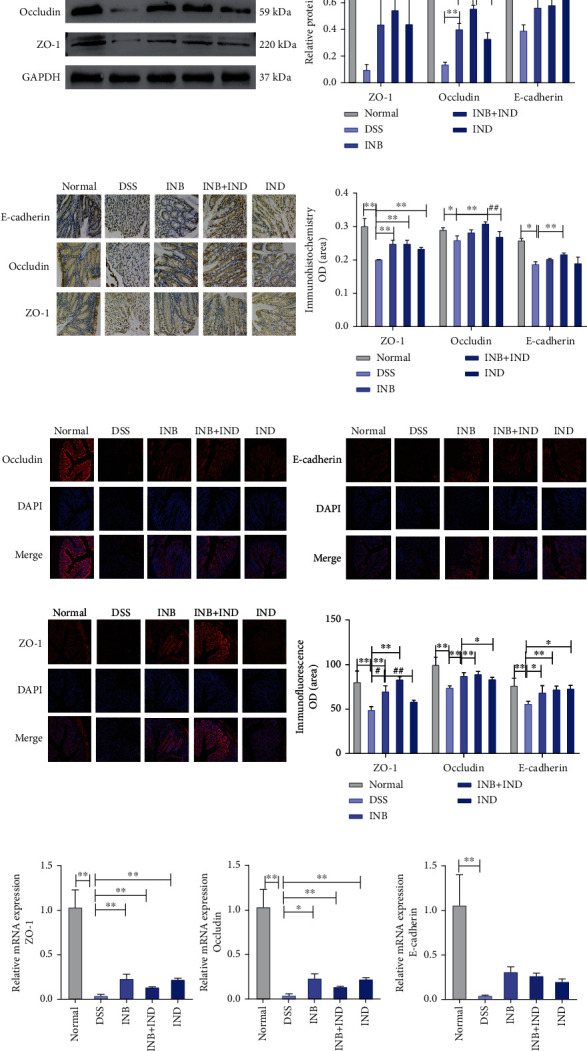
INB and IND maintained intestinal mucosal barrier function. (a) Representative image of western blot assay and the relative protein expression levels of tight junction proteins, E-cadherin, occludin, and ZO-1, *n* = 3. (b) Representative immunohistochemistry images and quantitative analysis of E-cadherin, occludin, and ZO-1, *n* = 3. (c) Representative immunofluorescence images and quantitative analysis of E-cadherin, occludin, and ZO-1, *n* = 3. (d) The mRNA expression level of tight junction proteins by real-time PCR, *n* = 3–5.

Tight junction proteins can protect the intestinal mucosa from potentially toxic substances, thereby maintaining the intestinal barrier. Therefore, the expression of tight junction proteins was evaluated by immunofluorescence and immunohistochemistry, and mRNA expression was assessed using real-time PCR. As shown in Figure 2(a), protein expression significantly increased in the colon tissue of the DSS group (*P* < 0.01). The combination of INB and IND induced a distinct decrease in occludin expression compared with INB or IND alone (*P* < 0.01), which indicated an enhanced effect after the combined treatment. Correspondingly, the immunohistochemical staining showed that the DSS group decreased the expression of tight junction proteins. Impressively, the occludin expression in the combination group was clearly higher than that in the INB or the IND group (Figure 2(b), *P* < 0.01). As displayed in Figure 2(c), either INB or IND increased the protein level of ZO-1, occludin, and E-cadherin, and the combination group caused a more substantial enhancement of the ZO-1 protein level than INB or IND alone (*P* < 0.01). Moreover, the mRNA expression of ZO-1 and occludin can be significantly enhanced after the three treatment groups (Figure 2(d), *P* < 0.01), while there were no significant effects on the mRNA expression of E-cadherin. Taken together, these data indicated that the combination treatment protects the intestinal mucosal physical barrier through upregulation of the expression of tight junction proteins.

### 3.3. INB and IND Regulated the Expression of MUC2 and Intestinal Permeability

**Figure 3:**
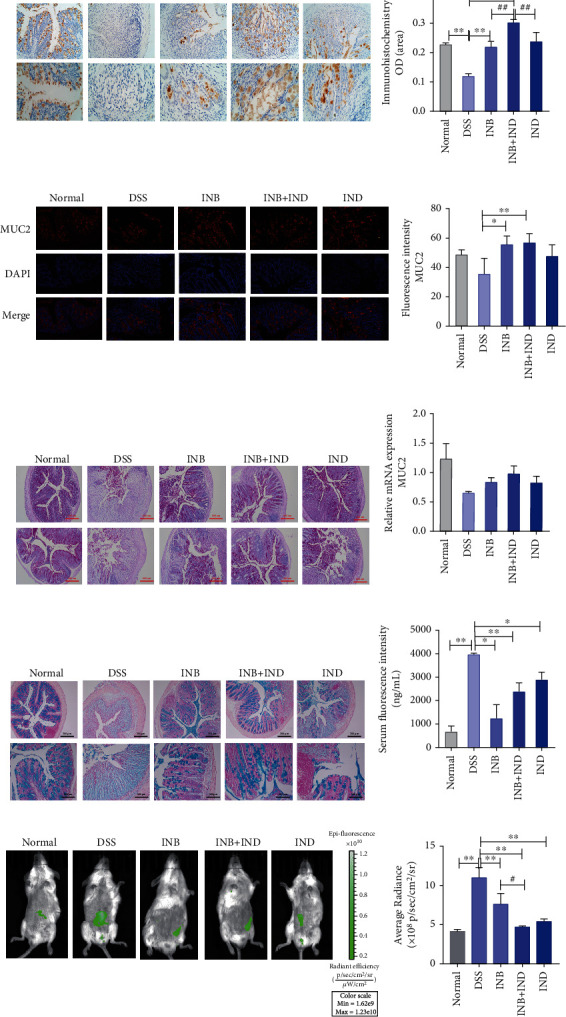
INB and IND regulated the expression of MUC2 and intestinal permeability. (a) Representative immunohistochemistry images and quantitative analysis of MUC2, *n* = 3. (b) Representative immunofluorescence images and quantitative analysis of MUC2, *n* = 3. (c) PAS staining of colon tissues, *n* = 3. (d) The mRNA expression level of MUC2, *n* = 3. (e) Alcian blue staining of colon tissue, *n* = 3. (f) Serum fluorescence intensity of FITC-dextran, *n* = 3. (g, h) Representative pictures of fluorescent imaging with FITC-dextran and the statistical analysis of the fluorescence intensity, *n* = 3. ^∗^*P* < 0.05 and ^∗∗^*P* < 0.01 compared with the DSS group; ^#^*P* < 0.05 and ^##^*P* < 0.01 compared with the INB+IND group.

MUC2, secreted from goblet cells, is particularly prominent in protecting the intestinal epithelium. To confirm the expression of MUC2 in the colon tissues, we further performed immunohistochemistry and immunofluorescence analysis. As shown in Figure 3(a), the MUC2 expression was dramatically upregulated in the treated group compared to the DSS group (*P* < 0.01). Moreover, in contrast to INB or IND alone, a significant increase in MUC2 protein expression was observed in the combination group (*P* < 0.01). However, the immunofluorescence analysis showed no statistically significant difference between the DSS and the normal groups (Figure 3(b)). The treatment group can upregulate the mRNA expression of MUC2 while no significant difference was observed (Figure 3(d)). As the first line of the intestinal barrier, the mucus layers can maintain the intestinal homeostasis by defending the intestinal epithelium against physical and chemical injuries. Therefore, goblet cells and mucus in the colon tissues were determined by Periodic Acid-Schiff (PAS) staining and Alcian blue. As shown in Figures 3(c)–3(e), loss of goblet cells and mucus was observed in the DSS group and was improved after the treatment, which was in accordance with the results of the MUC2 expression. An in vivo FITC-dextran permeability assay was conducted to evaluate intestinal permeability. The result showed that the treatment group downregulated the retention of fluorescent signals more than the DSS group (Figures 3(g) and 3(h), *P* < 0.01), which were further identified by the serum fluorescence intensity results (Figure 3(f), *P* < 0.01). Notably, the combination treatment showed significant effects on the serum fluorescence intensity than INB alone (*P* < 0.05), suggesting that the combination treatment may induce a more substantial effect on intestinal permeability. Collectively, these observations suggest that the INB and IND could regulate the expression of MUC2, and the intestinal permeability, especially in the combination group, appears to play a more critical role.

### 3.4. INB and IND Maintained the Mucosal Immune Homeostasis

**Figure 4:**
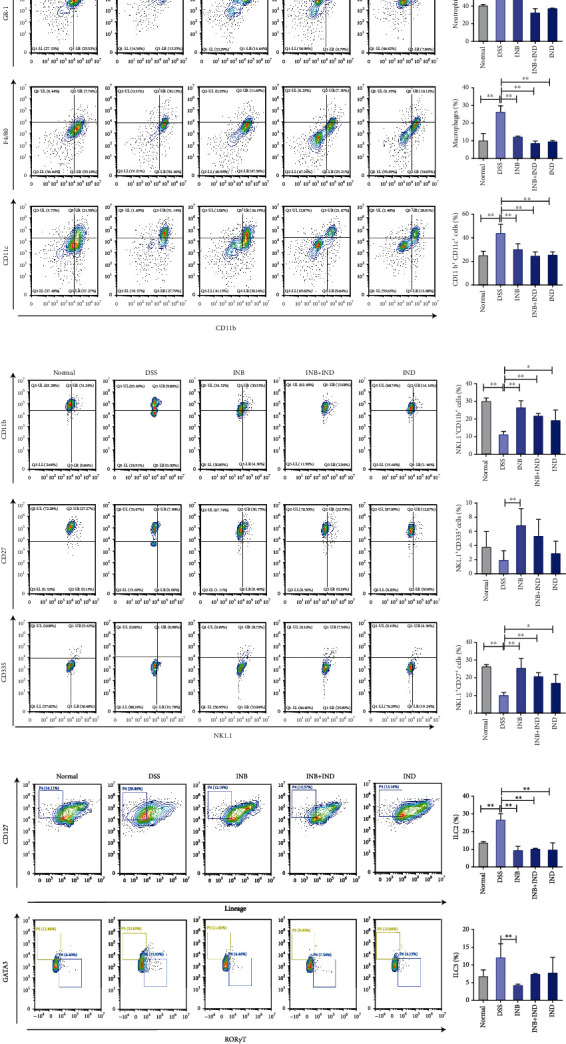
INB and IND maintained the mucosal immune homeostasis. (a) Flow cytometry and quantitative analysis of neutrophils (CD11b^+^ Gr-1^+^), macrophages (CD11b^+^ F4/80^+^), and dendritic cells (CD11b^+^ CD11c^+^), *n* = 3–5. (b) Flow cytometry and quantitative analysis of the proportion of activation of NK cells (CD335, CD27, and CD11b, gated on CD3^–^NK1.1^+^), *n* = 3–5. (c) Flow cytometry and quantitative analysis of the proportion of ILC2 (GATA3^+^) and ILC3 (ROR*γ*T^+^) in colon lamina propria, *n* = 3–5. ^∗^*P* < 0.05 and ^∗∗^*P* < 0.01 compared with the DSS group; ^#^*P* < 0.05 and ^##^*P* < 0.01 compared with the combination group.

ILCs and monocytes are essential for maintaining mucosal homeostasis and modulating immunity to intestinal infections [33]. Thus, cells were isolated from the lamina propria and were identified by flow cytometry. As shown in Figure 4(a), the analysis revealed that the populations of neutrophils (CD11b^+^ Gr-1^+^), macrophages (CD11b^+^ F4/80^+^), and dendritic cells (CD11b^+^ CD11c^+^) were significantly increased in the DSS group when compared with the normal group, consistent with the result of cytokine secretion in colon tissue (Figures 1(d)–1(i)). In contrast to the DSS group, a significant reduction in neutrophils (CD11b^+^ Gr-1^+^) occurred in the combination group and INB- and IND-alone groups (*P* < 0.01). Compared with the combination group, the INB group was less able to regulate neutrophils (*P* < 0.05). Further, we observed a significant decrease in dendritic cells (CD11b^+^ CD11c^+^) and macrophages (CD11b^+^ F4/80^+^) in the treatment group relative to the DSS group (*P* < 0.01). Notably, the IND group seemed to exhibit the most substantial regulation of dendritic cells though there was no significant difference between the treatment groups. The natural killer (NK) cells, including CD335 and CD11b, gated on CD3^–^NK1.1^+^, significantly decreased in the DSS group (*P* < 0.01) when compared with the normal group, which were similar to those obtained by Zhou et al. [34] and Saginbaeva and Lazebnik [35]. Notably, compared with the DSS group, a statistically significant increase in the relative quantity of the CD335^+^ and CD11b^+^ NK cells was observed after the treatment (*P* < 0.01), indicating that the therapeutic effect may be related to the activation of NK cells (Figure 4(b)). Among the treatment groups, the INB group appeared to have the most substantial effect on NK cells, which could modulate the level of NK cells to the normal group. As shown in Figure 4(c), we observed a significant decrease of ILC2 cells after the treatment of the combination group compared with the DSS group (*P* < 0.01), which was in accordance with the reduction of inflammatory cytokines. Furthermore, the INB group caused a more substantial suppression effect on ILC3 than the combination group or INB alone, indicating that the INB group may play a crucial role in ILC3 development. In short, these results indicated that the treatment groups decreased the infiltration of inflammatory cells into the lamina propria, and the IND group showed the strongest regulation, followed by the combination group and the IND group.

### 3.5. INB and IND Regulated the Intestinal Microbiota

**Figure 5:**
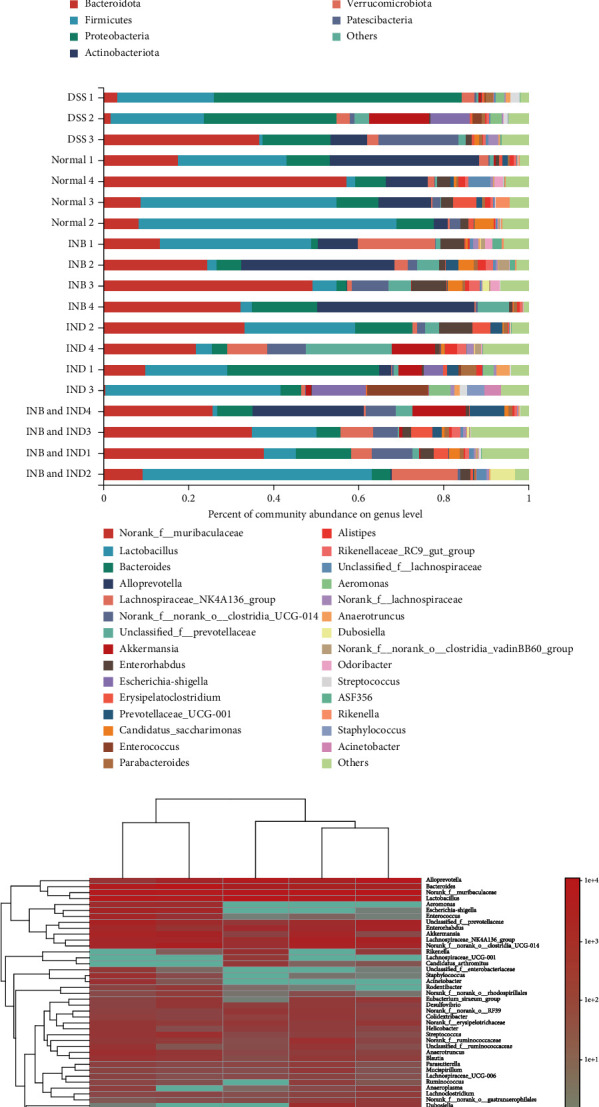
INB and IND regulated intestinal microbiota. (a) The Venn diagram of OTUs in each group. (b, c) The detailed composition and abundance at the phylum and the genus level. (d) Community heat map at the genus level. (e) Principal coordinate analysis (PCoA) and statistical analysis. (f) Nonmetric multidimensional scaling (NMDS) and statistical analysis. ^∗∗^*P* < 0.01 compared with the DSS group, *n* = 3.

The intestinal microbiota plays an essential role in mucosal homeostasis by maintaining the intestinal mucosal barrier integrity [36]. Therefore, we examined the effects of INB and IND on intestinal microbiota composition and functionality by performing a 16S rRNA sequencing analysis in the fecal samples of the mice. As shown in Figure 5(a), the OTUs in the DSS, normal, INB, IND, and combination groups were 302, 339, 328, 331, and 342, respectively. The result indicated that the microbiota diversity decreased in the DSS group, which the combination treatment could improve.

Next, the detailed composition and abundance in phylum and genus levels were illustrated as a heat map of sample clustering in Figures 5(b) and 5(c). At the phylum level, the most abundant phylum in all samples was Bacteroidota, Firmicutes, Proteobacteria, Verrucomicrobiota, and Actinobacteriota. Relative to the normal group, the abundance of Proteobacteria and Verrucomicrobiota increased in the DSS group, indicating that DSS treatment induced intestinal microbiota dysbiosis [37] (Figure 5(b)). Proteobacteria can produce endotoxin and are considered the primary pathogenic bacterium. With the increase of Proteobacteria abundance, the balance between pathogenic bacteria and protective symbionts is broken. Proteobacteria with adhesion and invasion ability potentially take advantage of genetic defects in pathogen recognition and bacterial clearance, causing inflammation and leading to the imbalance of microbiota and the mucosal immune system [38]. With the increase of Proteobacteria abundance, the balance between pathogenic bacteria and protective symbionts is broken. As beneficial bacteria, Firmicutes can produce metabolites to maintain the intestinal barrier and mucosal immunity and play an essential role in intestinal homeostasis [39]. Firmicutes increased in the administration group, indicating that they can play a therapeutic role by maintaining the intestinal barrier and mucosal immunity. Firmicutes in the intestinal microbiota of UC decrease, thereby increasing the risk of mucosal immune system disorders. Induced by DSS, the structure of intestinal microorganisms changes, which is related to the pathogenesis of UC [40].

At the genus level, the most abundant genera in all samples were *norank_f_Muribaculaceae*, *Lactobacillus|bacterioides*, *Alloperovella*, and *Lachnospiraceae_NK4A136_group* (Figure 5(c)). The *norank_f_Muribaculaceae* and *Lactobacillus* decreased, and *Bacteroides* increased in the DSS group compared to the normal group, consistent with previous research [41, 42]. The treatment group enriched the amounts of beneficial bacteria, like *norank_f_Muribaculaceae* and *Lactobacillus*. Additionally, the combination treatment and INB alone displayed an increase in the relative abundance of *Alloprevotella*. According to reports, *norank_f_Muribaculaceae* can reduce inflammation, inhibiting harmful bacteria and oxidative stress, and improve intestinal mucosal inflammation [43]. *Lactobacillus* can reduce proinflammatory cytokines, promote the increase of goblet cells and the secretion of antimicrobial peptides, and increase the level of the acetate to regulate the immune system and maintain the intestinal barrier function [44]. Therefore, INB and IND may treat ulcerative colitis by regulating the gut microbiota. The community heat map at the level was shown in Figure 5(d), and the dominant genus was different between the DSS and normal groups, which was in accordance with the results presented above.

**Figure 6:**
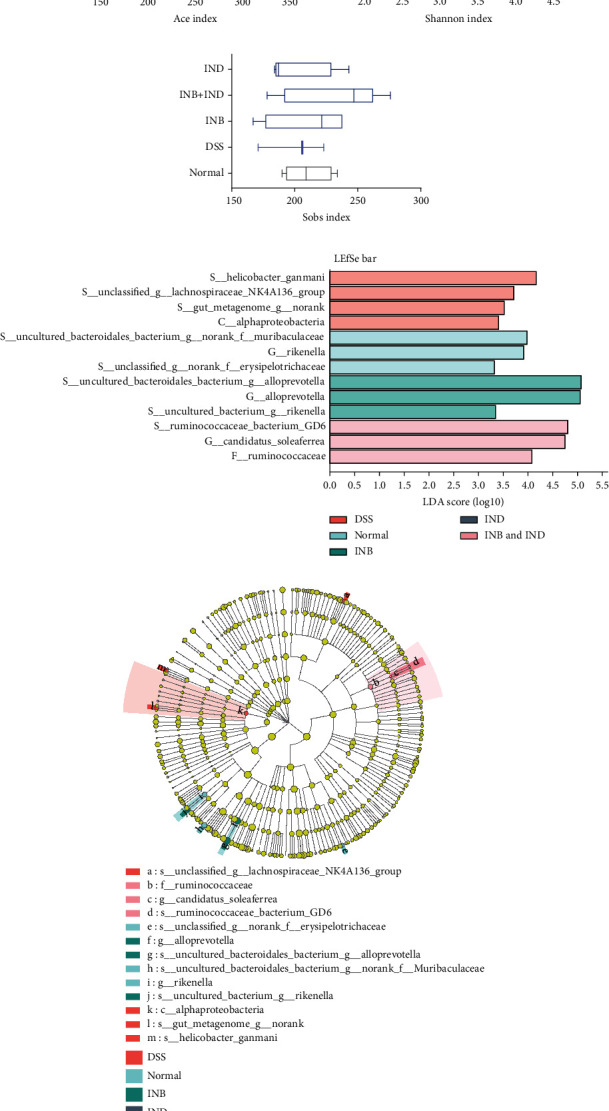
(a) Ace index of the sample. (b) Shannon index of the sample. (c) Sobs index of the sample. (d) LEfSe analysis of intestinal microbiota in each group, LDA score threshold > 3. (e) Cladogram.

Moreover, principal coordinate analysis (PCoA) (Figure 5(e)) and nonmetric multidimensional scaling (NMDS) (Figure 5(f)) analysis showed that the intestinal microbial landscape was markedly distinct in all groups, indicating differences in the intestinal microbial composition among the groups. The richness and diversity of the intestinal flora were evaluated by the alpha diversity analysis (Figures 6(a)–6(c)). The ACE index, Shannon index, and Sobs index in the combination group increased, while there was no significant difference between these groups. Linear discriminant analysis effect size (LEfSe) was used to assess differences in microbial communities between groups (Figures 6(d) and 6(e)). The DSS group showed enriched s_Helicobacter_ganmani, s_unclassified_g_Lachnospiraceae_NK4A136_group, s_gut_metagenome_g_norank, and c_Alphaproteobacteria. The dominant microbiota in the normal group was s_uncultured_Bacteroidales_bacterium_g_norank_f_Muribaculaceae, g_Rikenella, and s_unclassified_g_norank_f_Erysipelotrichaceae. The dominant microbiota in the INB group was s_uncultured_Bacteroidales_bacterium_g_Alloprevotella, g_Alloprevotella, and s_uncultured_bacterium_g_Rikenella. The dominant microbiota in the combination group was s_Ruminococcaceae_bacterium_GD6, g_Candidatus_Soleaferrea, and f_Ruminococcaceae. Briefly, the findings indicated that the treatment groups could modulate the intestinal microbiota composition.

### 3.6. Correlation Analysis between Intestinal Microbiota and Pathological Abnormities

**Figure 7:**
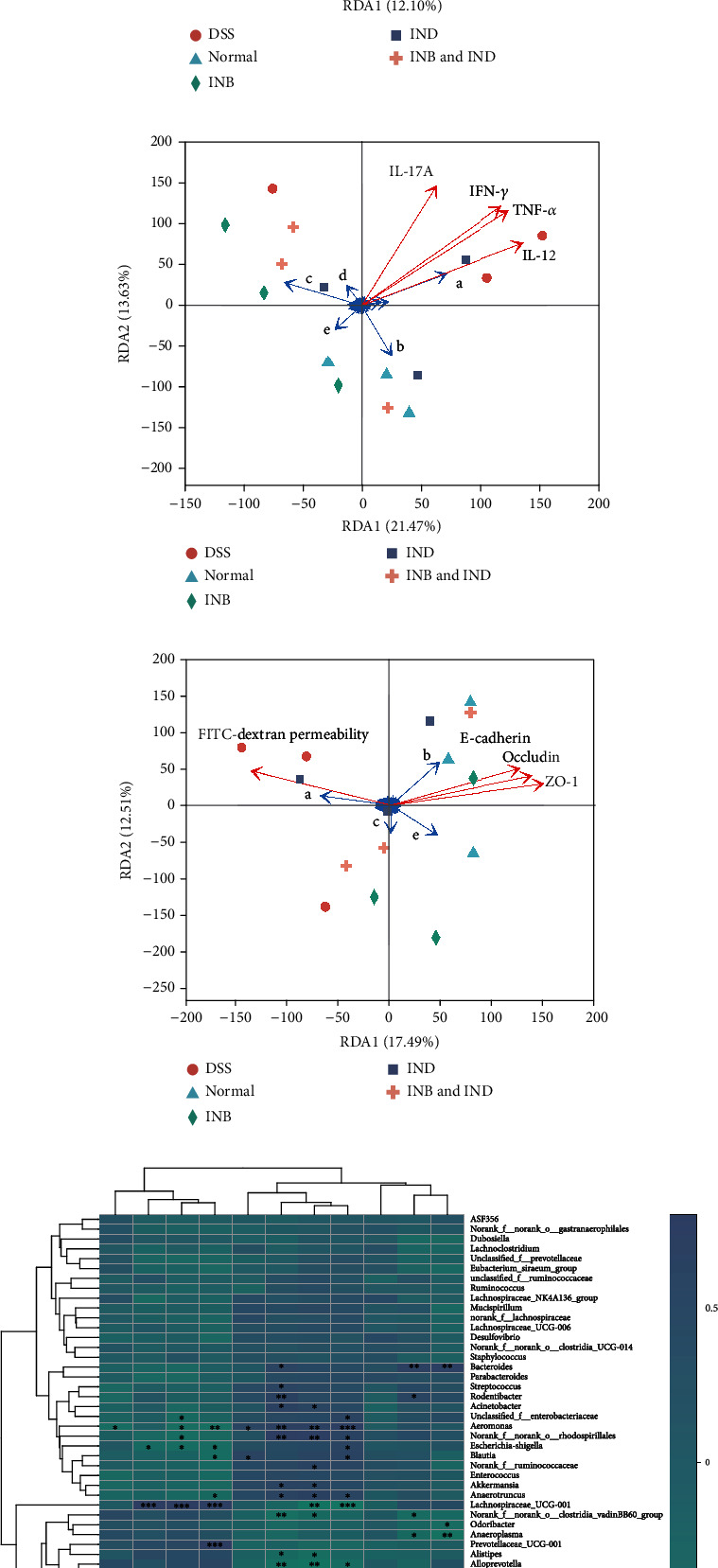
(a) Correlation among NK cells (CD335), ILC2, ILC3, and intestinal microbiota structure displayed by RDA/CCA. (b) Correlation among TNF-*α*, IL-12, IL-17A, IFN-*γ*, and intestinal microbiota structure displayed by RDA/CCA. (c) Correlation among E-cadherin, occludin, ZO-1, FITC-dextran permeability, and intestinal microbiota structure displayed by RDA/CCA. (d) Spearman correlation heat map of NK cells (CD335), ILC2, ILC3, TNF-*α*, IL-12, IL-17A, IFN-*γ*, E-cadherin, occludin, ZO-1, FITC-dextran permeability, and intestinal microbiota. A: *Bacteroides*; B: *Lactobacillus*; C: *norank_f_Muribaculaceae*; D: *norank_f_norank_o_Clostridia_UCG-014*; E: *Alloprevotella*; F: *Firmicutes*.

A Spearman correlation analysis was conducted to explore the relationships between intestinal microbiota and other pathological abnormities. Based on the redundancy analysis/canonical correspondence analysis (RDA/CCA) (Figures 7(a)–7(c)), *Bacteroides* were positively associated with ILC2, IL-12, TNF-*α*, IL-17A, IFN-*γ*, and FITC-dextran permeability, while being negatively associated with macrophages, ILC3, E-cadherin, occludin, and ZO-1. *Lactobacillus* was positively correlated with IL-12, E-cadherin, occludin, and ZO-1 while being negatively correlated with IFN-*γ*, IL-17A, and FITC-dextran permeability. *Norank_f_Muribaculaceae* was negatively associated with ILC2, TNF-*α*, IL-12, IL-17A, E-cadherin, occludin, ZO-1, and FITC-dextran permeability. *Norank_f_norank_o_Clostridia_UCG-014* was positively associated with IL-17A, IFN-*γ*, and TNF-*α*. *Alloprevotella* was positively associated with E-cadherin, occludin, and ZO-1, while being negatively associated with TNF-*α*, IL-12, IL-17A, IFN-*γ*, and FITC-dextran permeability. Furthermore, the result of the Spearman correlation heat map was consistent with the above results (Figure 7(d)). In conclusion, these results manifested that the intestinal microbiota homeostasis may be related to tight junction proteins, cytokines, immune cells, and intestinal permeability, indicating that these factors may function as a whole to modulate ulcerative colitis.

## 4. Discussion

UC, an incurable inflammatory disease, has been attributed to the complex interplay between intestinal microbiota, permeability, and mucosal immunity [45, 46]. The gut can produce MUC2 and form a mucus layer that is firmly attached to the gut epithelium and is generally bacteria free. However, some intestinal bacteria, such as *Akkermansia muciniphila* and *Enterorhabdus mucosicola*, can degrade intestinal mucus and multiply on the intestinal mucus layer, which increases bacterial penetration of the mucus layer leading to direct exposure of intestinal epithelial cells to the intestinal flora. Such harmful enterobacteria can directly affect intestinal structure and function, impair intestinal barrier function, and trigger inflammation and ulceration [47, 48].

The intestinal mucosal barrier, formed by the epithelial cells and the junctional complexes, including tight junctions, plays a critical role in the pathogenesis of UC [41, 49]. The inflammatory infiltrates in UC patients are limited to the colonic mucosa and are accompanied by severe infiltration of effector T cells with plasma cells [50]. Evidence suggests that both inflammatory and immune responses influence the intestinal mucosal barrier. In the intestinal mucosa of patients with UC, the cytotoxic effect of interleukin 13 produced by T cells on intestinal epithelial cells can alter the composition of tight junction proteins, which in turn alter intestinal mucosal penetration and ultimately affect the intestinal mucosal barrier [51]. In this study, we demonstrated that the combination treatment modulated mucosal homeostasis by regulating the expression of tight junction proteins, intestinal permeability, and intestinal microbiota.

Immune and inflammatory pathways of the intestinal epithelial barrier are essential parts of UC pathogenesis, and inflammatory cytokines can significantly modulate the disease progression of UC. It manifests in the fact that inflammatory cytokines can cause epithelial damage followed by intestinal barrier defects, so inhibiting the progress of inflammation can prevent the continued damage of the intestinal mucosal barrier. The essence of TNF-*α*, IL-12, and IL-23 inhibitors used in the treatment of UC is to inhibit the development of inflammation, prevent intestinal epithelial cell damage, and maintain the mucosal barrier [51, 52]. Accumulative evidence reveals that INB can exert remarkable anti-inflammatory effects in various ways and play a fundamental role in UC [25, 46]. In the present research, we found that the combination treatment could remarkably prevent mucosal inflammatory responses, leading to improved drug efficacy (Figure 1). We found that the combination treatment could increase the expression of ZO-1, occludin, E-cadherin, and MUC2, consistent with the improvement of intestinal permeability and mucosal inflammatory responses (Figures 2 and 3).

As an isomer of INB, IND plays a crucial role in immune regulation [26]. When harmful bacteria activate the innate immune system, the presentation of antigens by innate immune cells leads to T cell differentiation to produce IL-13 to destroy the epithelial cell barrier, followed by the impairment of mucin secretion function, and the intestinal mucosal barrier damage is exacerbated. Innate immune cells can present the antigen of harmful bacteria, leading to the differentiation of T cells to produce IL-13, damaging the intestinal mucosal barrier and further leading to increased intestinal permeability and enhanced absorption of bacterial products. Increased intestinal permeability and enhanced bacterial product absorption eventually trigger UC exacerbation. In addition, IL-37 will be produced when epithelial cells are damaged and inhibits innate immunity to exert anti-inflammatory effects to prevent further damage to the intestinal mucosal barrier by reducing TNF-*α* production by the lamina propria and IL-1*β* [53].

The prevalence of neutrophils, macrophages, and dendritic cells in the UC innate immune response is notable [54]. Dendritic cells and macrophages are able to not only present antigens to play a role in inflammation and innate immunity but also produce cytokines such as IL-12 and IL-23 under external stimuli. These cytokines can mediate the differentiation of CD4+ T cells into Th1 and Th17 cells. Th1 cells can secrete IFN-*γ* and TNF-*α*, and Th17 cells can secrete IL-17A and TNF-*α*, playing a role in intestinal mucosal inflammation and maintaining intestinal mucosal barrier homeostasis [53, 55]. Our study found that vast inflammatory cells (neutrophils, macrophages, and dendritic cells) appeared in the DSS group accompanied by the secretion of cytokines, which the combination treatment could alleviate. Although increasing evidence suggests that INB and IND could modulate regulatory T (Treg) cells [56], Foxp3^+^ T cells, and CD4^+^ T cells [24], it is still not known whether they could affect ILCs. ILCs, the most recently discovered group of immune cells, play a key role in defending against enteric pathogen invasion and maintaining mucosal immunity [57, 58]. It has been demonstrated that excessive ILC3 activation appears to aggravate experimental colitis by causing the release of IL-17A and IL-22 and an increase in neutrophil infiltration and tissue destruction in animal colitis models [59]. In the present research, we found that the combination treatment could substantially reduce the infiltration of ILC2 and ILC3, which accounted for the reduction of IL-22 and IL-17A and the increase of neutrophils.

ROS/RNS play critical roles in the pathogenesis of UC [60]. The excessive production of ROS/RNS can cause intense oxidative stress, leading to increased intestinal permeability, further disrupting the intestinal mucosal barrier [61]. The increased generation of ROS produced by immune cells may lead to tissue damage in UC [62]. In our study, we demonstrated that the administration of INB and IND could reduce the expression of ROS/RNS, which could contribute to the decreased intestinal permeability (Figure 1(j) and Figures 3(g) and 3(h)). The result could also be supported by the reduction of colon inflammation after INB and IND treatment (Figures 1(d)–1(i)). Consistently, the downregulation of immune cells, including neutrophils and macrophages, could account for the decrease of ROS/RNS. Hence, the administration of INB and IND attenuates DSS-induced UC via the enhancement of antioxidant functions. The intestinal microbiota consist of many microbes, which have emerged as a critical factor implicated in the development of mucosal homeostasis [63]. In addition, previous research showed that INB and IND could modulate intestinal microbiota [27]. Consistently, our results showed that the combination group existed with the most abundant intestinal microbiota, indicating that the diversity of intestinal microbiota can be maintained after treatment. In the present research, a decreased abundance of destructive bacteria and an increased population of probiotics, including *norank_f_Muribaculaceae*, *Lactobacillus*, and *Alloprevotella*, were observed after the treatment of the combination group (Figure 7). These probiotics are the biggest producers of short chain fatty acids (SCFAs) [64–66]. It has been proven that SCFAs could improve the recruitment of neutrophils in another UC model caused by 2,4,6-trinitrobenzenesulfonic acid (TNBS) [67] and were closely related to the tight junction protein complex [68]. Therefore, the combination treatment may involve immune cell activation and intestinal barrier maintenance by modulating the combination of intestinal microbiota.

Moreover, the complex interaction between immune cells and intestinal microbiota composition contributed to the pathogenesis of UC [69]. The commensal microbiota can mediate the development and function of specific lymphocyte subsets through the immune cells to molecule interactions and the host's signaling pathways. For example, Firmicutes and Bacteroidetes can produce short chain fatty acids from nondigestible carbohydrates. It improves the integrity of the intestinal mucosal barrier by regulating the intestinal immune response (T cells, neutrophils, and macrophages) and ultimately acts to prevent or ameliorate UC [70, 71]. It has been reported that the *Lactobacillus* genus could modulate immune cells by increasing the cytotoxicity of natural killer cells and phagocytosis of macrophages [72–74]. Earlier studies have shown that the Bacteroides may relate to increased intestinal permeability and inflammation [75], indicating that microbial metabolism may be crucial for modulating intestinal barrier responses. Furthermore, the abundance of *Norank_f_Muribaculaceae* was associated with intestinal inflammation [76]. Consistently, we found that *Lactobacillus*, *Bacteroides*, and *Norank_f_Muribaculaceae* were closely related to immune cells, tight junction proteins, cytokines, and intestinal permeability (Figure 7), from which we can deduce that the combination treatment may regulate the intestinal microbiota composition through immune cells and thus maintain the intestinal barrier.

## 5. Conclusions

In summary, our data indicate that combining INB and IND ameliorates DSS-induced colitis by reinforcing the intestinal barrier function. The underlying mechanism was related to the regulation of ROS/RNS and immune cells, including neutrophils, dendritic cells, NK cells, ILC2, and ILC3. Moreover, the mechanism may be related to modulating intestinal microbiota structure and composition. As promising treatments for UC, INB and IND provide a new direction for treating UC patients.

## Data Availability

The data used to support the findings of this study have not been made available because the research findings are commercialized.

## Supplementary Materials

Supplementary Materials The primers used for PCR amplification are shown in Supplementary Table 1.
