# Supplementary material for: Combination Therapy with Indigo and Indirubin for Ulcerative Colitis via Reinforcing Intestinal Barrier Function
Source: Oxid Med Cell Longev. 2023 Feb 14;2023:2894695. doi: 10.1155/2023/2894695 (PMC9943625; doi:10.1155/2023/2894695)
Supplement: Supplementary Materials — The primers used for PCR amplification are shown in Supplementary Table 1. [file 2894695.f1.pdf]

Supplementary Materials

Supplementary Table 1 shows the primers used for PCR amplification.

Table S1 The primers used for PCR amplification

Gene symbol	Forward sequence	Reverse sequence
GAPDH	GGTTGTCTCCTGCGACTTCA	TGGTCCAGGGTTTCTTACTCC
ZO-1	GCCGCTAAGAGCACAGCAA	TCCCCACTCTGAAAATGAGGA
E-cadherin	CAGGTCTCCTCATGGCTTTGC	CTTCCGAAAAGAAGGCTGTCC
Occludin	TTGAAAGTCCACCTCCTTACAGA	CCGGATAAAAAGAGTACGCTGG
MUC2	CGAGCACATCACCTACCACATCATC	TCCAGAATCCAGCCAGCCAGTC
